# Effect of external PEEP in patients under controlled mechanical ventilation with an auto-PEEP of 5 cmH_2_O or higher

**DOI:** 10.1186/s13613-016-0158-0

**Published:** 2016-06-16

**Authors:** Giuseppe Natalini, Daniele Tuzzo, Antonio Rosano, Marco Testa, Michele Grazioli, Vincenzo Pennestrì, Guido Amodeo, Francesco Berruto, Marialinda Fiorillo, Alberto Peratoner, Andrea Tinnirello, Matteo Filippini, Paolo F. Marsilia, Cosetta Minelli, Achille Bernardini, for the VENTILAB group, Filippo Albani, Maria Alfieri, Giovanni Amicucci, Barbara Buizza, Maria Giovanna de Cristofaro, Antonella Ferrari, Anna Granato, Irene Iannaco, Nicola Latronico, Umberto Lucangelo, Luigi Mendetta, Manuel Todeschini, Gabriele Tomasoni

**Affiliations:** Department of Anesthesia and Intensive Care, Fondazione Poliambulanza Hospital, Brescia, Italy; Department of Anesthesia and Intensive Care, Spedali Civili Hospital, Brescia, Italy; Department of Anesthesia and Intensive Care, SS. Annunziata Hospital, Savigliano, Italy; Department of Anesthesia and Intensive Care, Misericordia Hospital, Grosseto, Italy; Department of Anesthesia and Intensive Care, San Giovanni Bosco Hospital, Naples, Italy; Department of Anesthesia and Intensive Care, Agnelli Hospital, Pinerolo, Italy; Department of Anesthesia and Intensive Care, Santa Maria degli Angeli Hospital, Pordenone, Italy; Department of Anesthesia and Intensive Care, Cattinara Hospital, Trieste, Italy; Department of Anesthesia and Intensive Care, Mellino Mellini Hospital, Chiari, Italy; Department of Anesthesia, Critical Care Medicine and Emergency, University of Brescia at Spedali Civili, Brescia, Italy; Department of Anesthesia and Intensive Care, Cardarelli Hospital, Naples, Italy; Respiratory Epidemiology, Occupational Medicine and Public Health, Imperial College, London, UK

**Keywords:** Dynamic hyperinflation, Auto-positive end-expiratory pressure, Positive end-expiratory pressure, Flow limitation, Mechanical ventilation, Respiratory rate

## Abstract

**Background:**

In some patients with auto-positive end-expiratory pressure (auto-PEEP), application of PEEP lower than auto-PEEP maintains a constant total PEEP, therefore reducing the inspiratory threshold load without detrimental cardiovascular or respiratory effects. We refer to these patients as “complete PEEP-absorbers.” Conversely, adverse effects of PEEP application could occur in patients with auto-PEEP when the total PEEP rises as a consequence. From a pathophysiological perspective, all subjects with flow limitation are expected to be “complete PEEP-absorbers,” whereas PEEP should increase total PEEP in all other patients. This study aimed to empirically assess the extent to which flow limitation alone explains a “complete PEEP-absorber” behavior (i.e., absence of further hyperinflation with PEEP), and to identify other factors associated with it.

**Methods:**

One hundred patients with auto-PEEP of at least 5 cmH_2_O at zero end-expiratory pressure (ZEEP) during controlled mechanical ventilation were enrolled. Total PEEP (i.e., end-expiratory plateau pressure) was measured both at ZEEP and after applied PEEP equal to 80 % of auto-PEEP measured at ZEEP. All measurements were repeated three times, and the average value was used for analysis.

**Results:**

Forty-seven percent of the patients suffered from chronic pulmonary disease and 52 % from acute pulmonary disease; 61 % showed flow limitation at ZEEP, assessed by manual compression of the abdomen. The mean total PEEP was 7 ± 2 cmH_2_O at ZEEP and 9 ± 2 cmH_2_O after the application of PEEP (*p* < 0.001). Thirty-three percent of the patients were “complete PEEP-absorbers.” Multiple logistic regression was used to predict the behavior of “complete PEEP-absorber.” The best model included a respiratory rate lower than 20 breaths/min and the presence of flow limitation. The predictive ability of the model was excellent, with an overoptimism-corrected area under the receiver operating characteristics curve of 0.89 (95 % CI 0.80–0.97).

**Conclusions:**

Expiratory flow limitation was associated with both high and complete “PEEP-absorber” behavior, but setting a relatively high respiratory rate on the ventilator can prevent from observing complete “PEEP-absorption.” Therefore, the effect of PEEP application in patients with auto-PEEP can be accurately predicted at the bedside by measuring the respiratory rate and observing the flow-volume loop during manual compression of the abdomen.

## Background

Deciding whether to use positive end-expiratory pressure (PEEP) in mechanically ventilated patients with auto-PEEP is a daily challenge for intensivists, since in these patients the application of PEEP can increase or not the end-expiratory lung volume and end-expiratory plateau pressure [1, 2]. In some patients, here referred to as “complete *PEEP*-*absorbers,*” the application of PEEP reduces the auto-PEEP to maintain a constant total PEEP (i.e., the sum of PEEP and auto-PEEP as measured by the end-expiratory airway occlusion), therefore reducing the inspiratory threshold load and the work of breathing without detrimental cardiovascular or respiratory effects. In others, the total PEEP rises as a consequence of PEEP application and adverse effects can occur due to the worsening of hyperinflation. The impact of total PEEP in mechanically ventilated patients becomes relevant when end-inspiratory hyperinflation or hemodynamic impairment occurs, whereas end-expiratory hyperinflation could affect inspiratory threshold load and efficiency of respiratory muscles in patients with spontaneous respiratory activity.

Flow limitation occurs when the expiratory flow cannot be increased despite the raise of alveolar pressure, as usually occurs during the increase of an expiratory effort [1]. From a pathophysiological point of view, application of PEEP at a level lower than the patient’s auto-PEEP (e.g., 80–90 %) is expected to reduce auto-PEEP and leave total PEEP unchanged in the presence of flow limitation, while in the absence of flow limitation the applied PEEP should add to auto-PEEP and increase total PEEP [1, 3–5]. To the best of our knowledge, however, there is no clinical evidence confirming that flow limitation by itself is sufficient to prevent the increase in total PEEP when PEEP lower than auto-PEEP is applied. Indeed, the role of other factors that might predict a PEEP-absorber behavior, related to either patient characteristics (elastance, airway resistance, acute or chronic lung damage) or ventilatory setting (tidal volume, expiratory time, respiratory rate, minute ventilation), has never been studied. Moreover, the presence of flow limitation was not systematically assessed in previous clinical studies investigating the effect of applied PEEP on total PEEP and auto-PEEP in patients with respiratory failure. From these studies, we only know that, on average, total PEEP (or end-expiratory lung volume) increased by an amount lower than the applied PEEP when this was lower than auto-PEEP [5–14].

This study aimed to empirically assess the extent to which flow limitation alone explains a “complete PEEP-absorber” behavior (i.e., absence of further hyperinflation with PEEP), and to identify other factors associated with it. Moreover, we wanted to analyze the diagnostic performance of the model predicting the “complete PEEP-absorber” behavior which could improve the decision of how to use PEEP in patients with auto-PEEP.

## Methods

From January to June 2013, in a network of 11 Italian intensive care units, we performed a pre–post clinical trial where all patients were studied before and after PEEP application. Patients were considered for inclusion in the study if they met all of the following criteria: (1) age ≥18 years; (2) tracheal intubation (or tracheotomy) with controlled mechanical ventilation; (3) absence of any sign of spontaneous respiratory activity (absence of triggering, passive inspiration and passive expiration, as evaluated by airway pressure and airflow waveforms); (4) persistence of expiratory flow at the beginning of each inspiration; (5) no contraindication to compression of the abdomen; (6) absence of cardiovascular instability (mean arterial pressure >60 mmHg, systolic arterial pressure <180 mmHg, heart rate >40/min and <150/min); (7) arterial oxygen saturation >90 %; and (8) intracranial pressure <20 mmHg. All patients satisfying these criteria and with auto-PEEP ≥5 cmH_2_O at zero end-expiratory pressure (ZEEP) were enrolled in the study.

The primary aim of the study was to identify variables independently associated with “complete PEEP-absorber” behavior, i.e., unchanged total PEEP after application of PEEP equal to 80 % of auto-PEEP. Total PEEP was considered unchanged if its value increased up to 1 cmH_2_O, which represents the accuracy level of the pressure measurement. In a secondary analysis, patients who were not “complete PEEP-absorber” were classified as “high PEEP-absorber” if the increase of total PEEP was less than 50 % of applied PEEP; otherwise, they were classified as “low PEEP-absorber.” This created a three-level response variable, and the analysis was repeated to identify variables independently associated with “high PEEP-absorber” and with “complete PEEP-absorber” versus “low PEEP-absorber” response.

### Ethics, consent and permissions

The protocol was approved by the Institutional Ethical Committee (Comitato Etico ASL Brescia), and informed consent was obtained from patients or their next of kin, as appropriate.

### Measurements at baseline and after PEEP application

After enrollment, patients received volume-controlled ventilation with constant inspiratory flow while maintaining the tidal volume, respiratory rate and inspiratory time set by the attending physician. PEEP was set at 0 cmH_2_O. Three end-expiratory and three end-inspiratory airway occlusion maneuvers, each lasting 4 s, were then performed with at least ten uninterrupted breaths between maneuvers. Peak airway pressure (*P*_pk_), end-inspiratory plateau pressure (*P*_plat_), total PEEP (PEEP_tot_), tidal volume and inspiratory flow were measured. The mean value of each variable was used for subsequent analysis and calculation. Compliance of the respiratory system was calculated as tidal volume/(*P*_plat_ − PEEP_tot_) and resistance of the respiratory system as (*P*_pk_ − *P*_plat_)/inspiratory flow. *We calculated auto*-*PEEP* as the difference between PEEP_tot_ and applied PEEP [1].

After the occlusion maneuvers, the presence of flow limitation was assessed with manual compression of the abdomen [15–17]. The investigator put one hand gently on the patient’s abdomen, with the palm on the umbilicus, perpendicular to the axis between the xiphoid process and the pubis. After a short period, which allowed for recognition of the expiratory phase, the investigator exerted firm but gentle compression of the abdomen in an antero-posterior direction as soon as the insufflation was finished. This compression was maintained throughout expiration. The flow-volume loops obtained during passive expiration and during manual compression of the abdomen were superimposed, and flow limitation was diagnosed when all or part of the expiratory flow during manual compression of the abdomen and passive expiration was superimposed on the flow-volume loops. Three maneuvers were performed, and patients were classified as flow limited if flow limitation was confirmed in all three maneuvers.

We then applied a PEEP equal to 80 % of the patient’s auto-PEEP measured at ZEEP, while maintaining all other ventilator settings equal to those at baseline, and all measurements were repeated.

### Data validation

Each enrolled patient was assessed for reliability of measurements and absence of spontaneous respiratory activity. Data were considered reliable if the difference between each of the three measurements and their average value was lower than 10 % (a difference of 1 cmH_2_O was tolerated) for all airway pressure variables. Furthermore, the investigators captured images of the airway and flow waveforms during ventilation and during end-inspiratory and end-expiratory occlusions, and of the superimposed flow-volume loops obtained during manual compression of the abdomen and during passive expiration. These images were assessed and discussed by four senior authors (GN, DT, AR and MT), who had to confirm the absence of any sign of respiratory activity and the diagnosis of flow limitation. Only patients with data satisfying these validation criteria were included in the analysis.

### Statistical analysis

The size of the study was decided based on considerations on the number of predictors to be tested in the predictive model. Flow-limited expiration has been reported in approximately 40 % of patients with auto-PEEP [18], and we expected a similar percentage of “complete PEEP-absorber.” We anticipated that the enrollment of 100 patients would give about 40 events, allowing us to evaluate up to eight explanatory variables in a logistic model with “complete PEEP-absorber” as the outcome variable [19].

Data are shown as mean and standard deviation, median and interquartile range, or count and percentage, as appropriate.

#### Primary analysis (“complete PEEP-absorbers” vs. other patients)

In the univariate analyses, the variables to be tested were selected a priori and differences between groups were analyzed using logistic regression. All variables with a *p* value lower than 0.05 were included in a multiple logistic regression model to assess their independent association with “complete PEEP-absorber” behavior, with their effect expressed as odds ratio (OR) with 95 % confidence interval (95 % CI). Multicollinearity in the regression models was assessed by the variance inflation factor (VIF). Variables with VIF higher than 5 were removed one by one from the model, beginning from the covariate with the highest VIF.

Variables showing statistical significance in the multiple regression model were kept in the final predictive model. We performed internal validation using tenfold cross-validation to investigate model overfitting and correct for it. The diagnostic performance of our predictive model after cross-validation was evaluated in terms of discrimination, using the area under the receiver operating characteristics (ROC) curve corrected for overoptimism, and calibration, assessed using the mean absolute error. We also evaluated sensitivity and specificity, as well as positive and negative predictive values.

#### Secondary analysis (“low” vs. “high” and “complete PEEP-absorbers”)

Overall differences across the three groups were analyzed with a one-way analysis of variance for normally distributed continuous variables and a Chi-squared test for binary and nominal data. Pairwise comparisons were made with Tukey’s test and Fisher’s test, respectively. All variables with a p value lower than 0.05 were included in a multinomial logistic regression model to identify the variables independently associated with “high PEEP-absorber” and “complete PEEP-absorber,” with “low PEEP-absorber” as the reference level.

A *p* value threshold of 0.05 was for used for statistical significance. Statistical analyses were performed using the R statistical software, version 3.1.2 (R Foundation for Statistical Computing, Vienna, Austria, http://www.R-project.org).

## Results

Of the 203 patients screened, 118 (58 %) had auto-PEEP equal or greater than 5 cmH_2_O at ZEEP and were enrolled in the study. Eighteen enrolled patients did not satisfy the requirements for data validation, leaving 100 patients who were included in the analysis.

Thirty-nine percent of patients had the diagnosis of chronic obstructive pulmonary disease, and 19 % were admitted for acute exacerbation of chronic obstructive pulmonary disease. Pneumonia was diagnosed in 24 % of enrolled subjects. Simplified Acute Physiology Score 2 was 50 (40–61) at admission in intensive care unit, and Sequential Organ Failure Assessment score was 7 (5–9) on the day of the study (median and interquartile range).

**Fig. 1:**
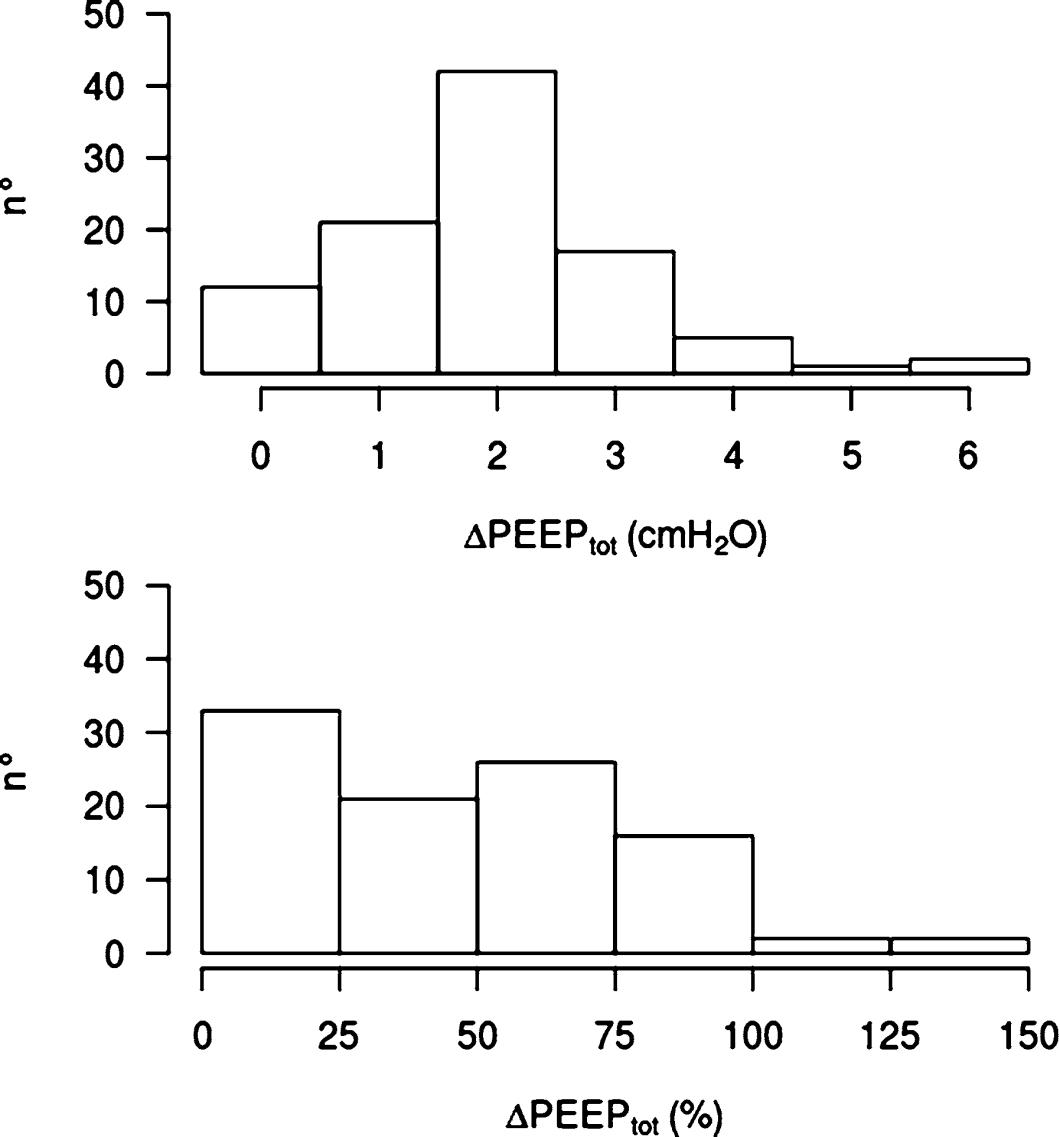
Frequency distribution of differences in total PEEP (ΔPEEP_tot_) in PEEP versus ZEEP phases. On the *upper side* the differences are shown as the absolute value in cmH_2_O; on the lower side the differences are expressed as a percentage of applied PEEP. *PEEP* positive end-expiratory pressure, *PEEP*_*tot*_ total PEEP, *ZEEP* zero end-expiratory pressure

Auto-PEEP at ZEEP was 7 ± 2 cmH_2_O, and the applied PEEP was 5 ± 1 cmH_2_O. On average, the addition of PEEP led to a total PEEP increased to 9 ± 2 cmH_2_O (*p* < 0.001). Thirty-three percent of patients were “complete PEEP-absorber,” 21 % were “high PEEP-absorber,” and the remaining 46 % were classified as “low PEEP-absorber.” The distribution of the post–pre difference in total PEEP (after application of PEEP as compared with baseline, ΔPEEP_tot_) across all patients is shown in the upper panel of Fig. 1. The lower panel of Fig. 1 shows ΔPEEP_tot_ expressed as a percentage of applied PEEP (e.g., 100 % if total PEEP increased by the full amount of applied PEEP; 0 % if the application of PEEP did not alter total PEEP). Figure 1 shows how changes in total PEEP after PEEP application vary across patients with auto-PEEP, with the majority of the patients showing changes in total PEEP that are halfway between those expected in the presence and absence of flow limitation.

The characteristics of complete, high and low PEEP-absorbers are compared in Table 1. The adjusted results of multiple logistic regression for the association between study variables and “complete PEEP-absorber” behavior are shown in Table 2. Expiratory time and minute ventilation were dropped from the model because of multicollinearity with respiratory rate. Respiratory rate and flow limitation were independently associated with “complete PEEP-absorber” behavior: Respiratory rate was inversely associated, whereas flow limitation was positively associated with the probability of being “complete PEEP-absorber.” The secondary analysis, in addition to confirming the association of respiratory rate and flow limitation with “complete PEEP-absorber” pattern, showed that the only characteristic associated with “high PEEP-absorber” was the presence of flow limitation (Table 3).

**Table 1:** Patients’ characteristics according to PEEP-absorber behaviors, classified as in the primary and secondary analyses

PEEP-absorber	*Primary analysis*: complete PEEP-absorber vs other patients			*Secondary analysis*: “other patients” classified as high and low PEEP-absorber (3-level variable)		
	Complete	Other	p	High	Low	p
Number of patients (%)	33 (33 %)	67 (67 %)	–	21 (21 %)	46 (46 %)	–
Total PEEP at ZEEP (cmH_2_O)	8 ± 3	6 ± 2	<0.001	8 ± 3	5 ± 1*^,#^	<0.001
Applied PEEP (cmH_2_O)	7 ± 2	5 ± 2	0.001	7 ± 2	4 ± 0*^,#^	<0.001
Total PEEP with PEEP (cmH_2_O)	9 ± 3	9 ± 2	0.65	10 ± 3	8 ± 1^#^	0.001
Female, n (%)	18 (55 %)	24 (36 %)	0.12	5 (24 %)	19 (41 %)	0.08
Age (years)	74 ± 8	70 ± 11	0.07	71 ± 10	69 ± 12	0.18
Body mass index (kg/m^2^)	30 ± 7	28 ± 6	0.05	31 ± 7	26 ± 5*^,#^	0.002
Respiratory rate (1/min)	16 ± 3	22 ± 4	< 0.001	20 ± 3	22 ± 4*	<0.001
Expiratory time (s)	2.7 ± 0.7	1.5 ± 0.5	< 0.001	1.7 ± 0.5	1.4 ± 0.5*	<0.001
Minute ventilation (l/min)	7.1 ± 1.4	10.5 ± 2	< 0.001	10 ± 2	11.1 ± 2.1*	<0.001
Tidal volume (ml/IBW)	8 ± 1	8 ± 1	0.94	8 ± 1.5	8 ± 1.2	0.29
Resistance (cmH_2_O∙l^−1^∙s)	21 ± 5	18 ± 7	0.05	20 ± 9	18 ± 6*	0.03
Elastance (cmH_2_O/l)	20 ± 5	19 ± 6	0.25	19 ± 6	18 ± 6	0.47
Flow limitation, n (%)	32 (97 %)	29 (47.8 %)	<0.001	19 (90 %)	10 (22 %)*^,#^	<0.001
Chronic pulmonary disease, n (%)	25 (76 %)	22 (33 %)	<0.001	12 (57 %)	10 (22 %)*^,#^	<0.001
History of smoking, n (%)	24 (75 %)	24 (38 %)	<0.001	9 (45 %)	15 (34 %) *	0.002
Acute pulmonary disease, n (%)	27 (84 %)	25 (37 %)	<0.001	12 (57 %)	13 (28 %)*	<0.001
PaO_2_/F_I_O_2_ (mmHg)	203 ± 82	275 ± 132	0.01	205 ± 89	306 ± 137*^,#^	<0.001
Supine position, n (%)	21 (64 %)	31 (46 %)	0.16	10 (48 %)	21 (46 %)	0.26

Figures are presented as number (percentage) or mean ± standard deviation, as appropriate

*PEEP* positive end-expiratory pressure, *IBW* ideal body weight

* *p* < 0.05 vs “complete PEEP-absorber”; ^#^ *p* < 0.05 versus “high PEEP-absorber”

**Table 2:** Multiple logistic regression: adjusted associations between study variables and “complete *PEEP*-*absorber*” behavior

	Odds ratio (95 % CI)	*p* value
Respiratory rate (min^−1^)	0.59 (0.42–0.76)	<0.001
Flow limitation	18 (1.7–476)	0.03
Resistance (cmH_2_O L^−1^ s)	0.94 (0.82–1.06)	0.29
Chronic pulmonary disease	3.2 (0.26–58)	0.38
Body mass index (kg m^−2^)	1.1 (0.94–1.2)	0.39
Acute pulmonary disease	2.3 (0.33–17)	0.39
History of smoking	2.8 (0.28–33)	0.39
PaO_2_/F_I_O_2_ (mmHg)	1 (0.99–1.01)	0.88

*CI* confidence interval

**Table 3:** Multinomial logistic regression: adjusted associations between study variables and “high PEEP-absorber” or “complete *PEEP*-*absorber*” behavior (“low PEEP-absorber” as reference level)

	Good PEEP-absorber		Complete PEEP-absorber	
	OR (95 % CI)	*p*	OR (95 % CI)	*p*
Flow limitation	20 (3.1–131)	0.002	76 (4–1425)	0.004
Respiratory rate (min^−1^)	0.91 (0.72–1.2)	0.46	0.56 (0.4–0.79)	0.001
PaO_2_/F_I_O_2_ (mmHg)	0.99 (0.98–1)	0.09	0.99 (0.98–1.01)	0.29
Body mass index (kg m^−2^)	1.1 (0.95–1.3)	0.2	1.1 (0.95–1.3)	0.16
Chronic pulmonary disease	3 (0.36–24)	0.31	7.3 (0.35–150)	0.2
Acute pulmonary disease	2.1 (0.31–13.9)	0.45	3.7 (0.34–41)	0.28
History of smoking	0.6 (0.07–5.2)	0.65	2 (0.12–33)	0.64
Resistance (cmH_2_O L^−1^ s)	1.01 (0.89–1.1)	0.93	0.94 (0.81–1.1)	0.38

*OR* odds ratio, *CI* confidence interval

The final predictive model to identify “complete PEEP-absorber” included flow limitation and respiratory rate, with the latter binarized as low and high using a threshold of 20/min; this corresponds to the threshold showing the best compromise between sensitivity and specificity to predict PEEP-absorber behavior in the ROC curve of the model with respiratory rate alone. The logistic regression equation of the model was: “complete PEEP-absorber” = −5 + 3.5 × respiratory rate <20/min + 2.9 × flow limitation. The model showed excellent overall predictive ability, with an area under the ROC curve of 0.92 (95 % CI 0.87–0.97). The tenfold cross-validation showed little evidence of overfitting in our predictive model. The high accuracy of the model was confirmed, with an area under the ROC curve corrected for overoptimism of 0.87 (95 % CI 0.79–0.95). The calibration of the model corrected for overoptimism was also good (mean absolute error = 0.03). Sensitivity, specificity and positive and negative predictive values are shown in Table 4, which also reports the diagnostic performance of two models with either flow limitation or respiratory rate alone. The model with both flow limitation and respiratory rate showed the best overall predictive ability, with high values across all indicators (ranging from 0.81 to 0.94).

**Table 4:** Diagnostic performance of the model to identify “complete *PEEP*-*absorber*” with different combinations of respiratory rate and flow limitation

	Sensitivity (95 % CI)	Specificity (95 % CI)	Positive predictive value (95 % CI)	Negative predictive value (95 % CI)
FL	0.97 (0.84–1)	0.57 (0.44–0.69)	0.52 (0.39–0.65)	0.97 (0.87–1)
RR < 20 min^−1^	0.91 (0.76–0.98)	0.85 (0.74–0.93)	0.75 (0.59–0.87)	0.95 (0.86–0.99)
RR < 20 min^−1^ and FL	0.88 (0.72–0.97)	0.9 (0.8–0.96)	0.81 (0.64–0.92)	0.94 (0.85–0.98)

*CI* confidence interval, *RR* respiratory rate, *FL* flow limitation

In practice, the data may be interpreted as follows: Flow-limited patients have approximately the same probability to be “complete PEEP-absorber” or not (positive predictive value: 0.52), but patients without flow limitation almost certainly are not “complete PEEP-absorber” (negative predictive value: 0.97). The probability to be “complete PEEP-absorber” is 0.75 when respiratory rate is lower than 20/min and increases to 0.81 when both flow limitation is present and respiratory rate is lower than 20/min. The absence of “complete PEEP-absorber” behavior can be accurately predicted based only on respiratory rate higher than 20/min (negative predictive value 0.95).

## Discussion

This study shows that the application of external PEEP is associated with an increase in total PEEP even in the presence of flow limitation, if the respiratory rate is sufficiently high. Respiratory rate and flow limitation, both measurable at the bedside, can predict whether PEEP application in patients with auto-PEEP is likely to result in an unchanged total PEEP rather than in an increased total PEEP with further hyperinflation. The predictive role of a low respiratory rate is novel, and the two factors together show accurate predictive ability, with an area under the ROC curve reaching 0.87. To our knowledge, this is the first study to develop a predictive model that could be used in clinical practice to guide the difficult choice of PEEP in patients with incomplete expiration.

As expected in patients with auto-PEEP ≥ 5 cmH_2_O, our study population showed a high percentage (65 %) of patients with chronic or acute lung disease, confirming a similar finding from a previous study in mechanically ventilated patients with acute respiratory failure [18]. Our sample is representative of those critically ill patients with at least 5 cmH_2_O of auto-PEEP, which have different characteristics when compared to the overall population of mechanically ventilated patients.

**Fig. 2:**
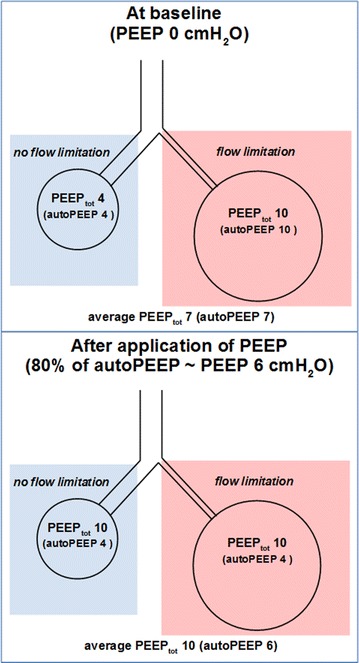
Effect of PEEP on areas with and without flow limitation. In the *upper part* an area without flow limitation with 4 cmH_2_O of auto-PEEP and a flow-limited area with auto-PEEP of 10 cmH_2_O at ZEEP are presented. Hypothesizing that these two areas evenly contribute to the expired volume, the average auto-PEEP of this model is 7 cmH_2_O. When PEEP of 6 cmH_2_O (about 80 % of auto-PEEP) is applied to the whole respiratory system (*lower part of the figure*), the part of the lung without flow limitation will increase its end-expiratory pressure by the same amount of the applied PEEP, then increasing total PEEP to 10 cmH_2_O, without any change in auto-PEEP. On the contrary, the flow-limited region is not expected to be further hyperinflated by a PEEP lower than its total PEEP, with the result that total PEEP does not change and auto-PEEP decreases. The average result of PEEP application on the whole lung will be a rise in total PEEP from 7 to 10 cmH_2_O: The two parts react to PEEP as either flow-limited or non-flow-limited areas, and the overall observed response to PEEP is intermediate between them. *PEEP* positive end-expiratory pressure; *PEEP*_*tot*_ total PEEP; *ZEEP* zero end-expiratory pressure

After application of PEEP equal to 80 % of the patient’s auto-PEEP, one-third of patients did not show an increase in total PEEP (“complete PEEP-absorber” behavior) and about half of patients showed an increase of less than 50 % of the applied PEEP. Most patients showed an increase in total PEEP that was halfway between that expected in the presence and absence of flow limitation. Apart from the possibility of decreased expiratory airway resistance and airway recruitment with PEEP [1], this behavior may be explained by the fact that flow limitation does not affect the lungs as a whole. Rather it should be regarded as a phenomenon occurring in some areas and not in others in the context of inhomogeneous lung disease [1, 20, 21]. As a consequence, within the same patient dynamic hyperinflation can occur in the absence of flow limitation in some lung regions, while it can be due to flow limitation in others. Therefore, it is not surprising that the increase in total PEEP exhibits a mixed behavior pattern, reaching the two extreme patterns of no change (“complete PEEP-absorber”) or increase by the same amount of the PEEP applied only in some patients. An explanatory model is reproduced in Fig. 2.

Our data show that flow limitation alone is not enough to predict a complete PEEP-absorber behavior, which requires the combination of flow limitation and low respiratory rate. In our study population, the threshold of 20/min used to define low respiratory rate was chosen as the best compromise between sensitivity and specificity in the ROC curve. This threshold, however, could be different in patients with characteristics or ventilator patterns different from those of the patients enrolled in our study.

The contribution of respiratory rate to “complete PEEP-absorber” behavior has a pathophysiological basis. The development of auto-PEEP in non-flow-limited areas becomes increasingly likely with the increase of respiratory rate and consequent reduction of expiratory time. Therefore, the application of PEEP in the presence of high respiratory rate could further worsen hyperinflation in non-flow-limited areas and prevent a “complete PEEP-absorber behavior.” Conversely, the reduction of respiratory rate can prevent auto-PEEP generation in the absence of flow limitation. In this case, all dynamic hyperinflation would be due exclusively to flow limitation, and the application of PEEP lower than auto-PEEP should not lead to further hyperinflation.

The prediction of the response to PEEP through evaluation of respiratory rate and flow limitation can be particularly useful when the measurement of total PEEP is not reliable or possible. This can occur when there is persistence of expiratory activity during end-expiratory occlusion [22–24], or when the mechanical ventilator does not allow end-expiratory occlusion. In addition, simply knowing that patients with high respiratory rate are not likely to be “complete PEEP-absorber” could be very important when flow limitation cannot be reliably assessed (e.g., during noninvasive ventilation in patients with respiratory failure) [25]: The use of PEEP should be cautious until the respiratory rate decreases, so that further hyperinflation can be avoided. Interestingly, while the presence of flow limitation and low respiratory rate could accurately predict a “complete PEEP-absorber” behavior, a “high PEEP-absorber” behavior was predicted with high accuracy by flow limitation alone. The clinical implication of this finding is that when PEEP is applied to a tachypneic flow-limited patient, we have in most cases an increase of total PEEP lower than half of the applied PEEP, whereas greater increases of hyperinflation are typical of patients without flow limitation.

In our study, the presence of flow limitation was assessed with the maneuver of manual compression of the abdomen, a simple technique validated in spontaneously breathing as well as sedated mechanically ventilated patients [15–17]. In mechanically ventilated patients, the manual compression of the abdomen has been shown to measure the lung volume at which flow limitation occurred with repeatability (i.e., variation among repeated measurements on the same subject under identical conditions) of 16 % and with a very good agreement with the technique of negative expiratory pressure (bias −0.16 ± 3.9 %; 95 % limits of agreement −7.8–7.5 %) [17]. In our study, we did not measure the lung volume at which flow limitation occurred, but simply we assessed whether flow limitation was present or absent. Therefore, we are confident that this approach was appropriate as a bedside evaluation of the presence of flow limitation. In situations where the maneuver of manual compression of the abdomen is impossible to perform or unreliable, it may be reasonable to assume that most of patients with chronic obstructive disease suffer flow limitation [18, 26].

### Study limitations

The choice to enroll only passive patients represents both a strength and a limitation of the study. The measurement of auto-PEEP was accurate, whereas it is challenging in actively breathing patients [8, 22–24]. However, our results should be generalized with caution to actively breathing patients, even if the breathing pattern of the patients in our study is similar to that observed in acute respiratory failure of different etiologies during assisted ventilation [27–29]. We cannot exclude that, in patients without flow limitation, total PEEP could be decreased by active expiration and hyperinflation could be actively limited at the expense of an increased effort of the expiratory muscles.

We tested the effects of applied PEEP equal to 80 % of auto-PEEP at ZEEP in patients with at least 5 cmH_2_O of auto-PEEP, and our results may not be generalizable to patients with lower auto-PEEP levels or in whom different levels of PEEP are applied. The individual response to PEEP depends on the level of PEEP applied, and in some patients, the “complete PEEP-absorber” behavior is observed only at PEEP levels lower than 80 % of their auto-PEEP [14].

Finally, we report findings obtained with the ventilator settings chosen by the clinicians. We did not investigate differences in the effect of PEEP application corresponding to different respiratory rates or tidal volumes in the same patient, and therefore, we cannot predict what would have been the response to PEEP if different ventilatory parameters had been used.

## Conclusions

When PEEP was applied to passive mechanically ventilated patients with auto-PEEP, one-third of patients did not increase total PEEP and about half increased it less than 50 % of the applied PEEP. We found that expiratory flow limitation was associated with both high and complete “PEEP-absorber” behavior, but our results also suggest that setting a relatively high respiratory rate on the ventilator can prevent from observing complete “PEEP-absorption.” Therefore, a simple evaluation of the patient’s ventilation showing high respiratory rate can allow to accurately exclude a “complete PEEP-absorber” behavior. Our study suggests that it is possible to predict at the bedside if the application of PEEP in patients with auto-PEEP can maximally reduce the inspiratory threshold load without any negative impact on hemodynamics and respiratory muscle mechanics. These findings could be used in routine clinical practice to help setting external PEEP when end-expiratory occlusion is not available or reliable.

## Abbreviations

OR: odds ratios
PEEP: positive end-expiratory pressure
*P*_pk_: peak airway pressure
*P*_plat_: end-inspiratory plateau pressure
ROC: receiver operating characteristics
ZEEP: zero end-expiratory pressure
